# Mevalonate supplementation to enhance euploid embryo formation in women of advanced maternal age: a study protocol for a randomized controlled trial

**DOI:** 10.1186/s13063-026-09522-9

**Published:** 2026-02-14

**Authors:** Shanshan Wang, Yonghong Zhang, Lingjuan Wang, Junshun Fang, Xiaoping Ye, Yanxin Sun, Haixiang Sun

**Affiliations:** 1 State Key Laboratory of Reproductive Medicine and Offspring Health, Center for Reproductive Medicine and Obstetrics and Gynecology, Nanjing Drum Tower Hospital, Clinical College of Nanjing Medical University, Nanjing, China; 2 Center for Molecular Reproductive Medicine, Nanjing University, Nanjing, 210000 China; 3 Changzhou Medical Center, Nanjing Medical University, Changzhou, 213000 China

**Keywords:** Mevalonate, Euploid embryos, Advanced maternal age, PGT-A, Randomized controlled trial

## Abstract

**Background:**

Oocyte aneuploidy is a major cause of pregnancy failure in women of advanced maternal age (AMA), and current assisted reproductive technologies offer limited effective strategies to prevent age-related decline in oocyte quality. The mevalonate (MVA) pathway in granulosa cells has recently been implicated in supporting meiotic maturation, but its therapeutic potential in humans remains unclear. This randomized controlled trial is designed to evaluate the effects of MVA supplementation on oocyte competence in women of AMA undergoing preimplantation genetic testing for aneuploidy (PGT-A).

**Methods:**

The study will be conducted as a single-center, prospective, randomized controlled trial. A total of 128 infertile women aged 38–42 years undergoing PGT-A will be enrolled and randomized to either the MVA supplementation group or the control group. Cumulus-oocyte complexes retrieved from each cycle will be allocated according to the randomization protocol, with an estimated 150 cycles analyzed. The primary outcome is the euploidy blastocyst rate, while the secondary outcomes are the clinical pregnancy rate and live birth rate following frozen embryo transfer (FET).

**Discussion:**

This trial is expected to evaluate the effects of MVA supplementation on euploidy blastocyst rate and clinical pregnancy outcomes in women of AMA undergoing PGT-A. These findings may provide valuable insights into the potential application of MVA supplementation as an adjunct treatment for infertility in this population.

**Trial registration:**

ClinicalTrials.gov NCT05788822. Registered in March 2023.

## Administrative information

Note: The numbers in curly brackets in this protocol refer to SPIRIT checklist item numbers. The order of the items has been modified to group similar items (see http://www.equator-network.org/reporting-guidelines/spirit-2013-statement-defining-standard-protocol-items-for-clinical-trials/).

**Table:** 

Title {1}	Mevalonate supplementation improved oocyte utilization in vitro in advanced maternal age: study protocol for a randomized controlled trial
Trial registration {2a and 2b}	ClinicalTrials.gov, ID: NCT05788822
Protocol version {3}	March 2023
Funding {4}	This work was supported by the Major Program of the National Natural Science Foundation of China (82030040), the Self-Determination Research Program of the State Key Laboratory of Reproductive Medicine and Offspring Health (SKLRM-2022D2), and the Jiangsu Frontier Technology R&D Program (Health and Wellness Sector) (BF2025626).
Author details {5a}	Shanshan Wang^1,2^, Yonghong Zhang^1,2^, Lingjuan Wang^1,2^, Junshun Fang^1,2^, Xiaoping Ye^1,2^, Yanxin Sun^1,2^, Haixiang Sun^1,2,3^* ^1^State Key Laboratory of Reproductive Medicine and Offspring Health, Center for Reproductive Medicine and Obstetrics and Gynecology, Nanjing Drum Tower Hospital Clinical College of Nanjing Medical University, Nanjing, China ^2^Center for Molecular Reproductive Medicine, Nanjing University, Nanjing, 210000, China ^3^Changzhou Medical Center, Nanjing Medical University, Changzhou, 213000, China
Name and contact information for the trial sponsor {5b}	Nanjing University, Hankou Road 22, Gulou District, Nanjing, Jiangsu Province, China, Postcode: 210093
Role of sponsor {5c}	The sponsor and funders were not involved in the study design and will not be involved in data collection, data analysis, or the drafting of the final report.

## Introduction

### Background and rationale {6a}

Oocyte aneuploidy is a major contributor to age-related fertility decline and a principal cause of pregnancy loss. Over the past decades, a global shift in reproductive behavior—most notably delayed childbearing—have increased the proportion of conceptions in older women and the incidence of age-associated fertility challenges [1]. Multiple factors contribute to these difficulties, among which declining oocyte quality—characterized by meiotic chromosome segregation errors—is a leading determinant and increases the risk of aneuploidy [2]. Epidemiological and clinical studies consistently show that the prevalence of aneuploid metaphase II oocytes rises sharply after the mid-30 s, with rates more than doubling in women beyond 34 years of age [3, 4]. Although postfertilization mitotic errors also contribute to chromosomal abnormalities, current evidence strongly supports that maternal age-related meiotic disturbances constitute the predominant origin of embryo aneuploidy [3, 5].

Proposed interventions to delay ovarian aging such as intraovarian stem-cell or platelet-rich plasma procedures remain investigational, and none has yet been proven in clinical trials to preserve oocyte genomic stability with advancing age [5, 6]. Assisted reproductive technologies (ART) including genetic testing for aneuploidy (PGT-A) permit selection of euploid embryos and inform transfer strategies but do not reduce the initial high frequency of aneuploid embryos seen in AMA. Current clinical guidelines advise PGT-A and frozen single embryo transfers for women with age-related fertility concerns [7]. However, embryo usability declines sharply after age 43 following PGT-A, underscoring the need for upstream biological interventions to improve oocyte quality at its source.

Emerging evidence implicates the mevalonate (MVA) pathway—a central metabolic route in cholesterol biosynthesis—in the regulation of meiotic maturation and genomic stability. As the primary regulator of cholesterol homeostasis, which undergoes age-related alterations [8], the MVA pathway supports oocyte maturation partly through luteinizing hormone receptor (LHR) and epidermal growth factor (EGR) signaling. Our prior investigations identified a marked suppression of MVA-related gene activity in granulosa cells from AMA patients compared to younger counterparts [8]. In animal models, exogenous MVA compounds have shown promise in correcting meiotic abnormalities and reducing aneuploidy rates in aging oocytes [8]. However, despite these mechanistic insights and preclinical data, the effect of MVA supplementation on euploid blastocyst formation in women of AMA remains unknown.

Given the potential role of MVA in restoring oocyte quality and the lack of effective interventions for women of AMA, this study proposes a randomized controlled trial to evaluate MVA supplementation during the pre-fertilization phase in women aged 38–42 undergoing PGT-A cycles, thereby facilitating clinical translation and potential integration into ART practice.

### Objectives {7}

To evaluate whether brief MVA supplementation in vitro prior to fertilization increases the yield of euploid embryos in women of AMA.

### Trial design {8}

This is a single-center, prospective, randomized, within-subject controlled superiority trial with a theoretical 1:1 allocation of cumulus-oocyte complexes (COCs) to intervention vs. control.

## Methods: participants, interventions, and outcomes

### Study setting {9}

Participants were recruited from the Center for Reproductive Medicine at Nanjing Drum Tower Hospital, China.

### Eligibility criteria {10}

***Inclusion criteria***

1. Women aged 38–42 years (inclusive) at the time of enrollment
2. Body mass index within the normal range (18.5–24.9 kg/m^2^)
3. Undergoing controlled ovarian hyperstimulation with either a short-acting agonist protocol or an antagonist protocol;
4. Scheduled for IVF/ICSI with PGT-A
5. Number of IVF/ICSI cycles ≤ 2

***Exclusion criteria***

1. Egg donation cycle
2. Known parental chromosomal abnormalities
3. Uterine disorders likely to impair implantation, including adenomyosis, uterine fibroids, uterine malformations, thin endometrium (< 7 mm), or endometriosis;
4. History of recurrent implantation failure (≥ 3 failed embryo transfers with good-quality embryos)
5. Severe systemic illness or uncontrolled endocrine/metabolic disease that may affect ovarian function (e.g., uncontrolled diabetes, thyroid dysfunction, or hyperprolactinemia)

Male partner eligibility is not restricted beyond a requirement for normal semen analysis; no additional exclusion criteria are applied to male partners. No restrictions are placed on study personnel or the participating center.

### Who will take informed consent? {26a}

Informed consent will be obtained by trained clinical research staff or the attending physician prior to any trial-related procedures. Participants will receive both written and verbal explanations of the study objectives, procedures, potential risks, and benefits. Sufficient time will be provided for questions and consideration. Written informed consent will be signed by each participant voluntarily.

### Additional consent provisions for collection and use of participant data and biological specimens {26b}

No additional consent will be required for the use of participant data beyond what is specified in the main consent form.

## Interventions

### Explanation for the choice of comparators {6b}

The standard control will consist of oocytes from the same individual that were cultured under routine IVF/ICSI laboratory conditions without MVA supplementation. This comparator represents the current standard of care in IVF/ICSI laboratory practice and allows for a direct evaluation of the effect of MVA treatment under otherwise identical conditions. By randomly allocating oocytes from the same patient into intervention and standard control groups, the design minimizes inter-individual variability and thereby enhances the internal validity of the study.

### Intervention description {11a}

A total of 150 PGT-A cycles will be included in the study. All retrieved COCs from each cycle will be randomly allocated into two parallel groups: the MVA supplementation group (intervention group) and the standard control group. Randomization will be performed at the level of sibling oocytes to minimize inter-individual variability.

#### Intervention group

COCs assigned to the intervention group will be incubated in fertilization medium supplemented with MVA metabolites at a concentration of 50 µM for 4 h prior to insemination. Following this exposure, all subsequent embryo culture procedures will be conducted according to standard IVF/ICSI protocols.

#### Standard control group

COCs in the standard control group will be cultured in standard fertilization medium without MVA supplementation for the same duration and under identical conditions as the intervention group. All laboratory procedures will be conducted under identical temperature, gas, and handling conditions to ensure consistency between groups.

#### Endometrial preparation

Endometrial preparation for frozen embryo transfer (FET) will be performed using hormone replacement therapy. From day 3 to day 16 of the menstrual cycle, patients will receive 4–6 mg/day of Femoston estradiol. Starting on cycle day 17, transvaginal ultrasound will be used to monitor ovulation status and evaluate endometrial thickness. Estradiol dosage will be adjusted according to individual endometrial response. Once the endometrium reaches a thickness of at least 8 mm, oral Femoston dydrogesterone combined with 60 mg of intramuscular progesterone will be administered daily for six consecutive days to complete luteal phase support.

#### Follow-up evaluation

**Fig. 1:**
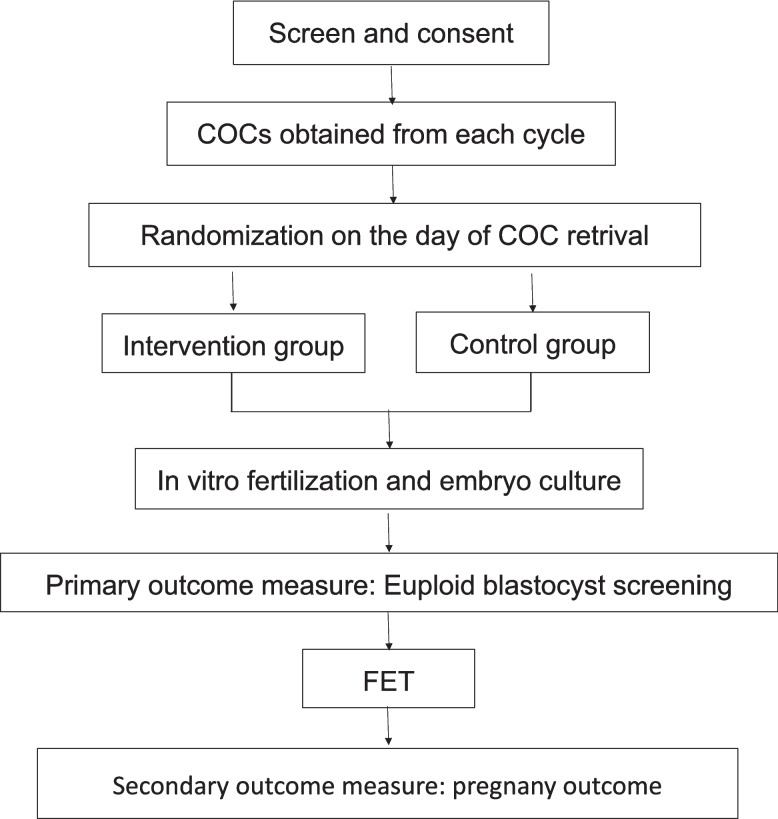
Flow chart of the study

1. Serum β-hCG levels will be measured 12 to 14 days following embryo transfer to confirm biochemical pregnancy.
2. Approximately 30 days post-transfer, a transvaginal ultrasound will be performed to detect the presence of an intrauterine gestational sac with a visible fetal heartbeat. The flow chart of this study is shown in Fig. 1.

#### Criteria for discontinuing or modifying allocated interventions {11b}

Participants will be excluded from continued follow-up if any of the following conditions occur:

1. Nonadherence to the prescribed treatment protocol
2. Unplanned discontinuation of the embryo transfer procedure
3. Administration of additional medications that could potentially influence oocyte and embryo development during the study period.

#### Criteria for study suspension

Study participation may be suspended under the following circumstances:

1. Voluntary request from the participant to discontinue
2. Safety concerns, including adverse events (AEs) that the investigator deems clinically significant and related to the intervention or any AE that the participant considers intolerable, regardless of severity
3. Inability to obtain complete follow-up data or loss of contact with the participant

### Strategies to improve adherence to interventions {11c}

During the RCT, participants will receive an incentive of reduced costs for euploid embryo tests.

### Relevant concomitant care permitted or prohibited during the trial {11d}

Any treatment or change outside of the study intervention will be monitored throughout the trial.

### Provisions for posttrial care {30}

No special provisions are offered.

### Outcomes {12}

#### Primary outcome measure

The primary outcome measure is the euploid embryo rate: the ratio of the euploid embryos to the number of mature oocytes.

#### Secondary outcome measures

Secondary outcome measures are the clinical pregnancy rate, miscarriage rate, and live birth rate.

- Clinical pregnancy rate: The ratio of the clinical pregnancy (intrauterine gestation sac with a fetal heart beat) number to the number of patients who received embryo transfer
- Miscarriage rate: The ratio of the number of nonviable pregnancies before 28 weeks of gestation to the number of clinical pregnancies
- Live birth rate: The ratio of the number of normal fetuses delivered after 28 weeks of gestation to the number of patients who received embryo transfer

### Participant timeline {13}

The participant timeline is shown in Table 1.

**Table 1:** Participant timeline

Timepoint	Study period							
	T1	T2	T3	T4	T5	T6	T7	T8
	Oocyte retrival	in vitro fertilization	Embryo culture	Euploid blastocyst screening	Day of embryo transfer	14 days after transfer	28/42 days after transfer	1 month postpartum
Eligibility screen	×							
Informed consent	×							
Basic information login	×							
Randomization		×						
Embryo quality			×		×			
Primary outcome measure				×	×			
Serum HCG level						×		
Secondary outcome measure							×	×
Compliance assessment				×	×			
Adverse events	×	×	×	×	×	×	×	×

### Sample size {14}

Among women of AMA (38 ≤ age ≤ 42 years) undergoing treatment at this center, the current euploid blastocyst rate (defined as the number of euploid blastocysts per mature oocyte retrieved) is approximately 10%. This study is designed to increase this euploid rate to 17% with a significance level (α) of 0.05. To achieve this, each group is expected to include 371 mature oocytes, accounting for an estimated 10% dropout rate. This results in a final target of 408 mature oocytes per group. Given the average mature oocyte yield per cycle in women aged 38–42 years is approximately 5.45 oocytes, the study anticipates enrolling approximately 150 cycles in total.

### Recruitment {15}

Participants will be recruited from Nanjing Drum Tower Hospital (Nanjing, China), a certified center for PGT. Recruitment efforts will involve multiple channels, including online announcements, hospital bulletin boards, and posters. A dedicated telephone line will be available for interested individuals to obtain additional information. Eligible candidates visiting the outpatient clinic will be invited to have an in-person discussion with study staff, during which comprehensive details about the trial will be provided. Patients who meet the inclusion criteria and express willingness to participate will be asked to sign a written informed consent form. The recruitment period is scheduled from December 2022 to December 2025, with an expected enrollment rate exceeding 95%.

## Assignment of interventions: allocation

### Sequence generation {16a}

All oocytes retrieved in each cycle will be evenly distributed into two fertilization dishes (Dish 1 and Dish 2). If an odd number of oocytes is retrieved, the extra oocyte will be placed in Dish 1. The randomization will be performed using SAS 9.4 statistical software, applying a completely randomized method.

### Concealment mechanism {16b}

The use of SAS to randomize using a password-restricted website will ensure concealment.

### Implementation {16c}

A research assistant independent from analysis generates the sequence and prepares dish labels.

## Assignment of interventions: blinding

### Who will be blinded {17a}

This study is an open trial. Both patients and researchers know the group assignments in the trial, and only the statisticians are blinded to the grouping.

### Procedure for unblinding if needed {17b}

No procedures are needed for unblinding until the finalization of the main data analysis.

## Data collection and management

### Plans for assessment and collection of outcomes {18a}

All clinical and laboratory data will be collected by trained staff using standardized forms. Intervention will be performed by experienced laboratory personnel using uniform criteria. To ensure data quality, double data entry and periodic cross-checks will be conducted. All data collection instruments have been validated internally and reviewed by the study monitoring team. Data collection forms will be stored electronically in a secured database and can be provided upon request or found in the supplementary materials.

### Plans to promote participant retention and complete follow-up {18b}

To enhance participant retention and ensure complete follow-up, all participants will receive regular reminders via phone or text messages. A dedicated study coordinator will be available to address participant concerns and facilitate scheduling of follow-up visits. Financial incentives, such as reduced PGT-A testing fees, will also be provided. Based on our experience, participants are usually grateful for any research conducted due to the strong reproductive requirements.

### Data management {19}

All clinical and laboratory data will be entered into a password-protected electronic data capture system by two independent research assistants to ensure accuracy (double data entry). Each participant will be assigned a unique study ID to ensure de-identification. Range checks, logical consistency checks, and regular audits will be performed to maintain data quality. All data will be stored on a secure hospital server with restricted access granted only to authorized study personnel. Backups will be scheduled weekly. Data will be encrypted both in transit and at rest.

### Confidentiality {27}

All personal information from potential and enrolled participants will be collected through secure hospital systems and documented using standardized case report forms. Each participant will be assigned a unique study identification code, and identifiable information will be stored separately from clinical data. Only authorized members of the research team will have access to identifiable information. All electronic data will be stored on password-protected, encrypted servers within the hospital’s network. Paper-based records, if any, will be kept in locked filing cabinets in restricted-access areas.

Throughout the trial and after its conclusion, all data will be handled in accordance with applicable data protection regulations. Identifiable data will not be shared outside the research team. Upon study completion, personal identifiers will be removed or anonymized before data archiving or analysis.

### Plans for collection, laboratory evaluation, and storage of biological specimens for genetic or molecular analysis in this trial/future use {33}

Trophectoderm biopsy samples collected during PGT-A will be used solely for chromosomal screening as part of the clinical protocol. No additional biological specimens will be collected for genetic or molecular studies. Biopsied cells will be analyzed using next-generation sequencing in a certified clinical laboratory. No biological samples will be stored for future use in ancillary studies. Participants will be informed accordingly, and no additional consent for biobanking is required.

## Statistical methods

### Statistical methods for primary and secondary outcomes {20a}

Data analysis will be conducted using SPSS version 13.0. Descriptive statistics will summarize baseline demographic and clinical characteristics. Categorical variables—including clinical pregnancy rate, implantation rate, and miscarriage rate—will be compared between groups using the chi-square (*χ*^2^) test or Fisher’s exact test where appropriate. Continuous variables such as patient age, baseline FSH levels, antral follicle count (AFC), total gonadotropin dosage and duration, number of oocytes retrieved, and fertilization rate will be analyzed using independent-samples *t*-tests or nonparametric equivalents as appropriate. To account for potential confounding factors, multivariable regression analyses will be conducted. Logistic regression models will be applied for binary outcomes (e.g., clinical pregnancy, implantation, miscarriage), while linear regression will be used for continuous outcomes (e.g., number of mature oocytes, fertilization rate). Models will adjust for maternal age, baseline FSH levels, AFC, ovarian stimulation parameters, and other relevant covariates. For pregnancy outcomes, paternal factors such as age and semen parameters will also be included as covariates. A two-sided *p*-value < 0.05 will be considered statistically significant. Effect sizes will be reported with 95% confidence intervals. Sensitivity analyses will be conducted to test the robustness of the results.

### Interim analyses {21b}

No interim analyses are planned for this study.

### Methods for additional analyses (e.g., subgroup analyses) {20b}

No additional analyses are planned.

### Methods in analysis to handle protocol nonadherence and any statistical methods to handle missing data {20c}

Data will be analyzed following the ITT principle. Missing data will be analyzed. The imputation of missing values is not intended, although multiple imputations might be used within sensitivity analyses assuming data are missing at random.

### Plans to give access to the full protocol, participant-level data, and statistical code {31c}

There are currently no plans to make the full protocol, participant-level dataset, or statistical code publicly available. However, de-identified summary data may be shared upon reasonable request to the corresponding author, subject to approval by the study team and ethics committee.

## Oversight and monitoring

### Composition of the coordinating center and trial steering committee {5d}

As this is a single-center study, the establishment of a formal coordinating center is considered unnecessary. The core research team holds weekly meetings to oversee trial progress and address operational matters, while the principal investigators participate in monthly meetings to review key developments and provide strategic guidance.

### Composition of the data monitoring committee and its role and reporting structure {21a}

A formal data monitoring committee has not been established for this study. As the trial is single center, non-blinded, and involves minimal risk to participants, the principal investigators and study team are considered sufficient to oversee data quality and participant safety. Any adverse events or protocol deviations will be reviewed during regular team meetings.

### Adverse event reporting and harms {22}

All AEs, whether solicited or spontaneously reported, will be monitored and recorded throughout the trial. Participants will be instructed to report any discomfort, unexpected symptoms, or complications during and after the intervention period. AEs will be assessed by the clinical research team for severity and potential association with the study intervention. Serious adverse events (SAEs) will be reported to the institutional ethics committee within 24 h of identification. All AEs and SAEs will be documented in the case report forms and the study database. If necessary, the principal investigator may suspend or discontinue the intervention for the affected participant.

### Frequency and plans for auditing trial conduct {23}

No formal external audits are planned for this study. Given that the trial is single center, investigator-initiated, and poses minimal risk, routine internal monitoring will be performed by the study team to ensure compliance with the protocol and data integrity. The institutional ethics committee retains the right to conduct audits at its discretion if necessary.

### Plans for communicating important protocol amendments to relevant parties (e.g., trial participants and ethical committees) {25}

Any important protocol amendments, such as changes to eligibility criteria, primary outcomes, or analysis methods, will be submitted for approval to the institutional ethics committee prior to implementation. Investigators will be informed through team meetings and written notices. If applicable, affected trial participants will be re-consented using updated informed consent forms. The trial registration record will be updated accordingly. Relevant regulatory bodies and scientific journals will be notified if the changes materially affect the study design or interpretation.

### Dissemination plans {31a}

The results of this trial will be disseminated through peer-reviewed journal publications and presentations at academic conferences. In accordance with trial registration policies, results will also be reported on the relevant clinical trial registry upon study completion. If requested, a lay summary of the findings will be provided to participants. There are no publication restrictions imposed by the sponsor.

## Discussion

AMA is strongly associated with oocyte aneuploidy, which remains a major cause of implantation failure, miscarriage, and reduced live birth rates. Emerging evidence has highlighted the MVA pathway as a key regulator of oocyte meiotic maturation and genomic stability. Previous studies demonstrated that disruption of this pathway in granulosa cells is associated with impaired LHR and EGF signaling, leading to defective meiosis and increased aneuploidy [8]. In animal models, supplementation with MVA metabolites has been shown to mitigate meiotic errors, restore proper chromosome segregation, and reduce aneuploidy rates in aging oocytes [8]. These preclinical findings suggest that maintenance of MVA pathway integrity is critical for safeguarding oocyte quality during reproductive aging. Building on this evidence, the present study represents the first randomized controlled trial (RCT) to evaluate MVA supplementation in human-assisted reproduction. By directly translating preclinical discoveries into a clinical setting, this trial seeks to test whether short-term MVA supplementation during the pre-fertilization stage can enhance oocyte competence and increase the yield of euploid embryos in women of AMA.

A RCT was selected as the study design as it represents the gold standard for clinical research, effectively minimizing bias and providing the highest level of evidence for causal inference. A within-subject controlled approach was further adopted, whereby COCs from the same stimulation cycle are randomly allocated to MVA-supplemented or standard fertilization medium. This strategy maximizes statistical efficiency by controlling for inter-individual variability and thereby enhances the ability to detect the true effect of MVA supplementation. Women aged 38–42 years were specifically targeted as younger patients show low baseline aneuploidy, while women above 42 typically yield insufficient oocytes for analysis. This choice is also consistent with clinical practice guidelines, which recommend considering preimplantation genetic screening in women over 38 years [9]. The intervention parameters (50 µM for 4 h before insemination) were chosen based on effective conditions in animal studies and are readily integrated into routine IVF/ICSI practice.

The primary endpoint, euploidy efficiency (euploid blastocysts per mature oocyte), was selected because it directly reflects meiotic fidelity and genomic stability. Secondary endpoints, including clinical pregnancy and live birth rates, were included to capture downstream clinical implications and to assess the potential translational impact of the intervention.

Several limitations should be acknowledged. First, it is open-labeled as the MVA-supplemented and standard media cannot be blinded; although this may introduce bias, the use of standardized procedures and an objective genomic endpoint minimizes subjectivity. Second, as a single-center trial, the generalizability of findings may be limited, though the intervention is simple and easily replicable. Third, the choice of euploidy efficiency as a surrogate endpoint enables feasibility with a smaller sample size but may not fully reflect clinical outcomes; therefore, pregnancy and live birth are included as secondary endpoints. Operational challenges include slow recruitment due to diminished ovarian reserve, strict standardization of intervention procedures, and potential attrition or missing data. These will be addressed through extended recruitment, detailed SOPs with staff training, and intention-to-treat analyses with retention strategies.

If effective, MVA supplementation could serve as a simple, low-cost upstream intervention that can be seamlessly integrated into standard IVF/ICSI protocols, potentially improving embryo usability and pregnancy outcomes in women of AMA. Scientifically, the trial will provide the first human evidence supporting the role of the MVA pathway in oocyte meiotic fidelity, thereby advancing understanding of reproductive aging mechanisms. Future research should investigate dose-response relationships, assess applicability in other populations such as younger women with diminished ovarian reserve, and conduct multicenter randomized trials powered for live birth outcomes to validate and extend these findings.

In summary, this trial evaluates a biologically grounded and operationally feasible intervention addressing the root cause of embryo aneuploidy in AMA. The results will inform both clinical practice and basic science, guiding next steps in optimizing fertility care for women of AMA.

## Trial status

The study was conceived and designed in 2022. This protocol version was approved by the Affiliated Drum Tower Hospital of Nanjing Medical University. It was registered on 28 March 2023, and the registry number is NCT05788822 (https://register.clinicaltrials.gov). The recruitment period is from March 2022 to May 2024. The last patient completed embryo biopsy in Jan 2025, and the last follow-up visit (including clinical outcome confirmation) will be completed in Dec 2025. The present manuscript was submitted in Sep 2025, prior to the end of data analysis. The delay in submission was due to the time required for complete embryo ploidy testing, clinical follow-up, and final data integrity verification. No interim or post hoc changes were made to the protocol after trial completion.

## Data Availability

All data generated or analyzed during this study are included in this article. Further enquiries can be directed to the corresponding author.

## Abbreviations

ART: Assisted reproductive technology (ART)
PGT-A: Preimplantation genetic testing for aneuploidy
MVA: Mevalonate
FET: Frozen embryo transfer
AMA: Advanced maternal age
COCs: Cumulus-oocyte complexes
RCT: Randomized controlled trial
SAEs: Serious adverse events
AFC: Antral follicle count
AEs: Adverse events
FET: Frozen embryo transfer
LHR: Luteinizing hormone receptor
EGR: Epidermal growth factor
ART: Assisted reproductive technologies
IVF: In vitro fertilization
ICSI: Intracytoplasmic sperm injection
